# A Multi Size-Level Assessment of Benthic Marine Communities in a Coastal Environment: Are They Different Sides of the Same Coin?

**DOI:** 10.1371/journal.pone.0129942

**Published:** 2015-06-15

**Authors:** Claudia Vannini, Marta Volpi, Claudio Lardicci

**Affiliations:** 1 Department of Biology, University of Pisa, Pisa, Italy; 2 Center for Geomicrobiology, Institute of Bioscience, University of Aarhus, Aarhus, Denmark; University of Waikato (National Institute of Water and Atmospheric Research), NEW ZEALAND

## Abstract

Organism body size has been demonstrated to be a discriminating element in shaping the response of living beings to environmental factors, thus playing a fundamental role in community structuring. Despite the importance of studies elucidating relations among communities of different size levels in ecosystems, the attempts that have been made in this sense are still very scarce and a reliable approach for these research still has to be defined. We characterized the benthic communities of bacteria, microbial eukaryotes, meiofauna and macrofauna in a coastal environment, encompassing a 10000-fold gradient in body size, testing and discussing a mixed approach of molecular fingerprinting for microbes and morphological observations for meio- and macrofauna. We found no correlation among structures of the different size-level communities: this suggests that community composition at one size-level could have no (or very low) influence on the community composition at other size-levels. Moreover, each community responds in a different way to the environmental parameters and with a degree of sensitivity which seems to increase with organism size. Therefore, our data indicate that the characterization of all the different size levels is clearly a necessity in order to study the dynamics really acting in a system.

## Introduction

Among the many features which can be related to species distribution and abundance, organism size has been recently shown to be very important. Indeed, organism body size has been demonstrated to be a discriminating element in shaping the response of living beings to environmental factors, thus playing a fundamental role in community structuring [1, 2, 3]. For example, Farjalla and colleagues [1] hypothesized that the influence of environmental conditions on community structuring is higher at higher size-levels. An explanation to this finding resides probably in the fact that body size is actually related to other aspects of species ecology. Indeed,organism size determines lifespan and reproductive rate and, as a consequence, the fitness of a population in a certain environment [4]. Dimensions have been also shown to directly influence the dispersion rate and, hence, the distribution of a certain species [2]. Therefore, density and abundance are affected as well [5], thus shaping the structure of communities for what concerns, for example, species richness [3]. The effects of body size on species ecology has been proved to be so wide that Woodward [6] proposed to measure it as a synthetic parameter encapsulating and condensing a large amount of biological information. Beside these considerations, organism body size is deeply linked to the trophic level of a certain species in the trophic chain [5, 7]. The need for research elucidating relations among different trophic levels in ecosystems is largely recognized. Studies focusing on multiple levels at the same time are particularly important for unraveling patterns of species diversity and their determining factors, but also for a better understanding of species interactions and for a better planning of conservation strategies [8, 9]. Taking this into account, research focusing on communities characterized simultaneously at different levels from the same environment can give a valuable contribution to elucidate both how organisms with different body size are affected by environmental factors and how different trophic levels interact with each other.

Despite the well-recognized importance of such studies [1], the attempts that have been made in this sense are still extraordinarily scarce. Indeed, research investigating the community structure at different levels represent still a low number and are frequently limited to specific taxonomic groups (see for example Lear and colleagues [10] or Doi and colleagues [9]). The reasons for this situation are of cultural, but also of methodological nature. Cultural, because such studies require knowledge on very different kind of organisms and, in most cases, the highly-specialized competences of many research groups lead their work toward highly-specialized research fields (i.e. on one specific taxonomic group). Methodological, because a multidisciplinary approach is absolutely essential for performing such studies and this requires skills on different techniques and procedures: investigations on microbes have to be necessarily performed in a different way with respect to those on bigger organisms. Therefore, a tested, efficient and reliable approach, able to guarantee a clear comparison among communities of different size levels, still has to be defined. Indeed, several different methods have been used in the past for the characterization of microbial communities and their comparison with those of bigger organisms. These methods comprehends Denaturant Gel Gradient Electrophoresis (DGGE [11]), Automated Ribosomal Intergenic Spacer Analysis (ARISA [12]), Terminal-Restriction Fragment Length Polymorphism (T-RFLP [2]) or, recently, Next Generation Sequencing (NGS) techniques like pyrosequencing [13, 14].

Most of studies realized up to now concern freshwater habitats [1, 2, 3, 9, 11, 12] and only a couple concerns different habitats: soil [13] and marine sediments and water [14]. As spatial scales have been shown to be of great importance in marine habitats [15], the marine environments constitute particularly interesting systems for studying community structure at different size levels.

In this paper we present the simultaneous characterization of the benthic communities of bacteria, microbial eukaryotes, meiofauna and macrofauna in a coastal environment of soft bottoms, encompassing a 10000-fold gradient in body size. We performed a comparative analysis, considering how the structure of these communities was shaped as a reply to the same environmental conditions, depth and sediment granulometry. In particular, we verified in the marine environment the hypothesis of increasing environmental determinism with increasing body size. Moreover, we tested and comparatively analyzed two different methods for the characterization of microbial communities, namely T-RFLP and LH-PCR, which was here applied for the first time also for the study of eukaryotic communities.

## Materials and Methods

### Study area

The investigated area is located along the shoreline of San Rossore, in the San Rossore—Massaciuccoli Natural Park. This park occupies 30 Km of flat coastal strip in northern–western Tuscany (Ligurian Sea), between the provinces of Pisa and Lucca. The shore of San Rossore is microtidal (astronomical tidal range of 35 cm), with sediments mainly composed by coarse sand. It is characterized by a prevalent northward distal and proximal drift and very exposed to the winds of the third quadrant [16]. The sampled area is influenced northward by a channel called "Fiume Morto", where bathing is permanently forbidden. The “Fiume Morto” carries to the sea treated wastewater and also direct discharges of the municipal territory placed on the north of the Arno river.

### Sampling design and methods

**Fig 1:**
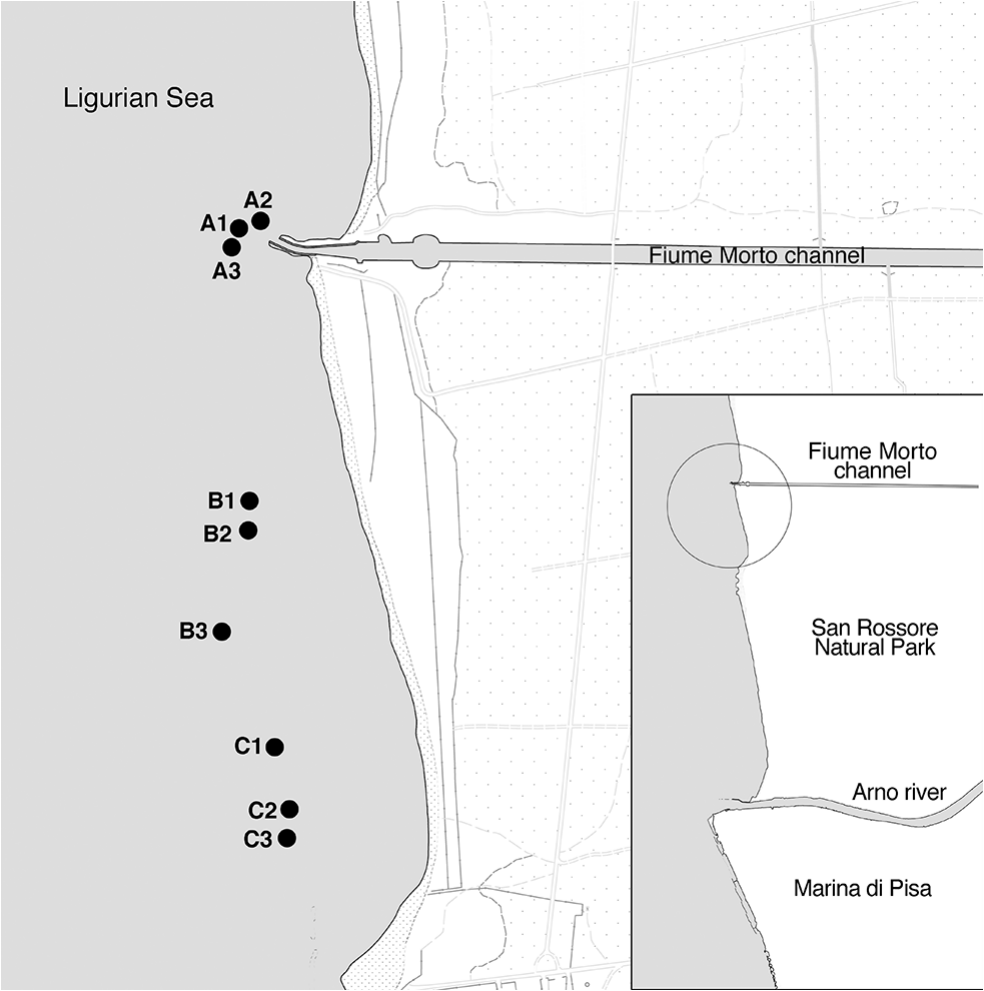
Study area and locations of the sampled sites. The map shows the study area and the locations of the sites; for each site, two detached replica were performed on a few meters distance. Geographical coordinates of each sampling site are reported in S1 Table.

The sampling plan was devised to make a comparison among the benthic community features of the different zones according to a hierarchical design, since communities often show differences in species composition and structural parameters in marine sediments at a variety of spatial scales [17]. Samples were collected at the end of May 2012 at about 150 meters from the coast and at a depth comprised between 3 and 4 m in order to sample the same sandy habitat. Three different areas were sampled: the first one, in correspondence of the mouth of the “Fiume Morto” (A), and two more areas on the south of the first one (B, C). Geographical coordinates of each sampling site are reported in S1 Table. In each area three sites were randomly selected and two detached replica were performed on a few meters distance (Fig 1). For each replica five consecutive samples were taken with the use of a Ekmann-Birge grab: the first one in order to collect sediment to analyze the bacterial community, the second one to collect sediment for analysis of eukaryotic communities, the third one for meiofauna analysis, the fourth for macrofauna analysis and the fifth for sediment granulometry and organic matter determination. In particular, meiofauna samples were subsampled at 4 cm depth in the collected sediment using a corer 3 cm inner diameter and immediately fixed in 4% formalin, while for sediment analysis the samples were frozen. Macrofauna replicates were preserved in 4% formalin after sieving at 0.5 mm in the field. Samples for bacterial and microbial eukaryote communities were stored in cool bags and brought to the laboratory a few hours after collection. Here they were immediately treated and stored for subsequent analysis. Salinity and temperature were measured close to surface and bottom with a Beckmann salinometer, while geographical coordinates and depth were taken with a GPS/DepthFinder Lowrance for each replicate.

### Analysis of bacterial and microbial eukaryotic communities

Samples were used for total genomic DNA extraction, according to the procedure described in the extraction protocol of the kit PowerSoil DNA Isolation Kit (MO BIO Laboratories, Inc.) and the presence of DNA molecules in the obtained solution was verified by an electrophoresis on agarose gel. 16S and 18S rRNA genes were used as genetic markers for the characterization of, respectively, bacterial and microbial eukaryotic communities. For T-RFLP technique, two different restriction enzymes (AluI and BsuRI, Fermentas, Canada, 0.2 μ/μl final concentration) were used on each sample, in order to better detect microbial diversity. Concerning LH-PCR technique, the choice of the region to be amplified was the point at issue. For bacteria, we inferred the V1 region (near to 5' end of the 16S rRNA gene), between position 8 and position 355 of *E*. *coli*, as reported in literature [18]. The LH-PCR technique has been applied to eukaryotic genes in this work for the first time. Therefore, we tested this technique on two distinct regions of the gene encoding for the ribosomial small subunit of eukaryotes: the V1 region and the V4 region, one of the most variable region within this gene [19, 20]. More details on analysis of microbial communities are reported in S1 File.

### Meiofauna, macrofauna and sediment analysis

The macrofauna was sorted in the laboratory and, where possible, identified to species level. After washing on a 63 mm sieve to remove formalin, the meiofauna was extracted using a colloidal silica solution (Ludox) with a specific gravity of 1.15. Meiofauna components were enumerated under a stereoscope. Small sizes and physical fragility make a precise species-level identification very difficult to reach for these organisms and the risk of a wrong classification is very high. Therefore, meiofauna components were only identified to major taxa.

Organic matter was determined by ignification at 450°C for four hours after removing excess carbonate by 10% HCl. Grain size distributions were determined by dry sieving and pipette analysis of the silt and clay fractions. The sediment fractions were defined according to the Wentworth scale [21].

### Statistical analysis

The peak heights obtained by T-RFLP and LH-PCR were standardized by equalising their sums among different runs with their average levels, and recalculated for each run. As a final step, peaks showing heights lower than 50 fluorescence units were excluded from the analysis.

To calculate and compare species diversity among samples, we calculated the number of Operational Taxonomic Units (OTU) detected in each sample.

For all the assemblages the distribution of samples was represented on a two-dimensional plane through non-metric multi-dimensional scaling (nmMDS) [22], based on Bray-Curtis similarity matrix after a square root data transformation.

One-way analysis of similarities (ANOSIM [23]) was used to test for significant differences among the three sampled areas. The pairwise comparison between different assemblages was made by means of the test RELATE, a comparative (Mantel-type) test on similarity matrices [24]. The same test was used in order to compare matrices obtained with different methods for the same microbial assemblage (i.e. different restriction enzymes and different LH-PCR regions) and matrices obtained from data at different taxonomic levels.

Environmental data were squared root transformed, standardized and normalized after performing data scatter plots of all pair-wise combinations of environmental variables to approximate normality. The surface salinity, the coarse sand fraction, the organic matter percentage, the depth and the longitude values that better discriminate the different areas were considered and ordinated using principal components analysis (PCA).

The relationships among all multivariate assemblage structures and combinations of abiotic data were analyzed using the BEST/BIO-ENV procedure [25] This method is based on rank correlation between a similarity matrix derived from biotic data and matrices derived from all combinations of environmental variables. It was used in order to define suites of variables that best explain the assemblage structures.

Statistical analysis were performed using the software PRIMER [26].

## Results

### Structure of different size-levels communities and comparative analysis among different areas

#### Bacterial community

**Fig 2:**
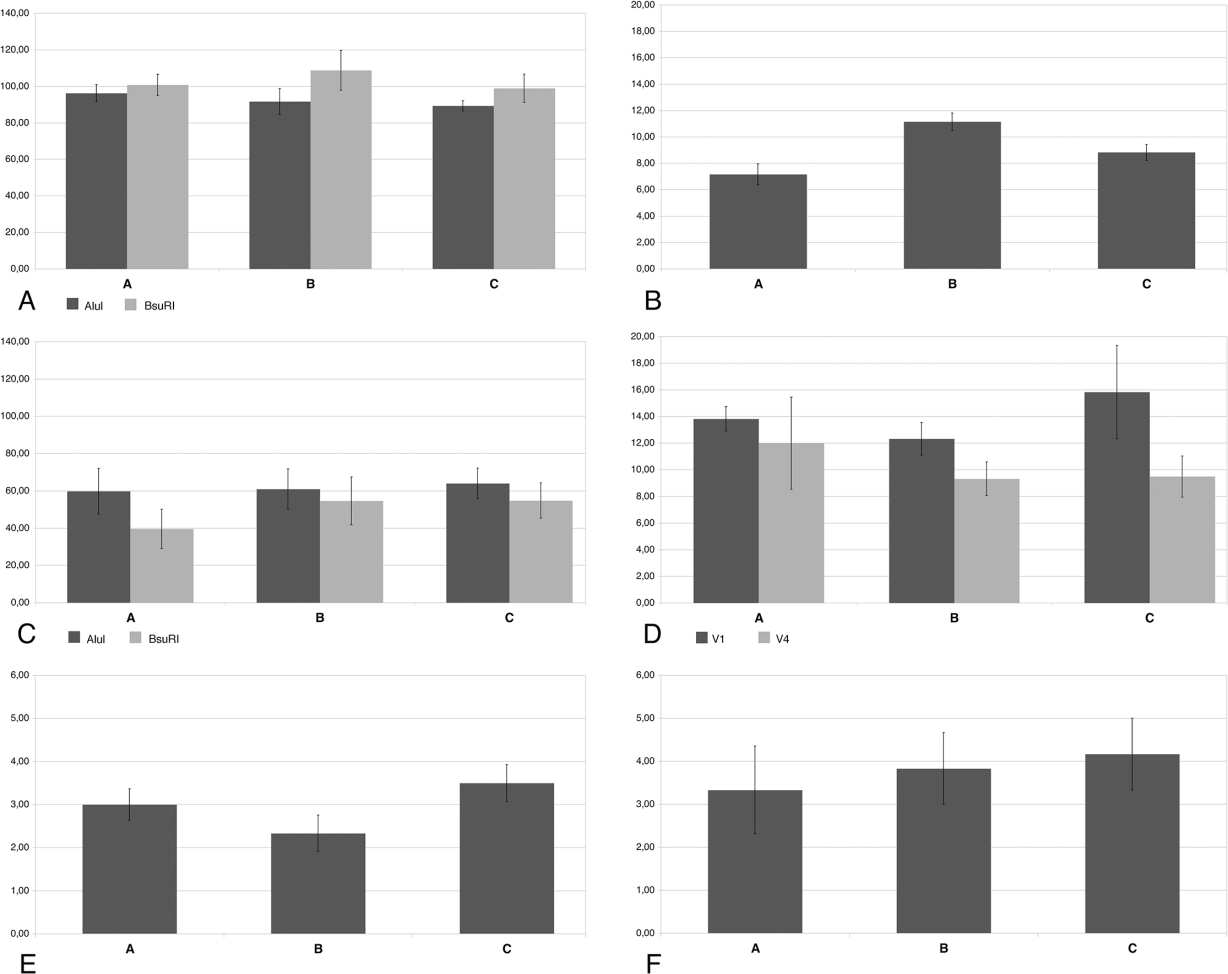
Number of taxa at the different size-levels. Mean total number of taxa of the four different size-level communities in the three areas (mean ± SE): bacterial OTUs assessed by T-RFLP (A) and LH-PCR (B); microbial eukaryotic OTUs assessed by T-RFLP (C) and LH-PCR (D); meiofauna taxa (E); macrofauna species (F).

For the bacterial community, the number of detected OTU in each sample was within a range of 70–114 with the enzyme AluI and 79–157 with the enzyme BsuRI, while the same number was much lower with the LH-PCR, in a range of 5–12. The average number of OTUs in area A, B and C obtained with the two enzymes is reported in Fig 2A. For the LH-PCR technique the average numbers are reported in Fig 2B. The difference in the percentage number of OTUs between T-RFLP and LH-PCR is 90.58% (±2.71%).

**Fig 3:**
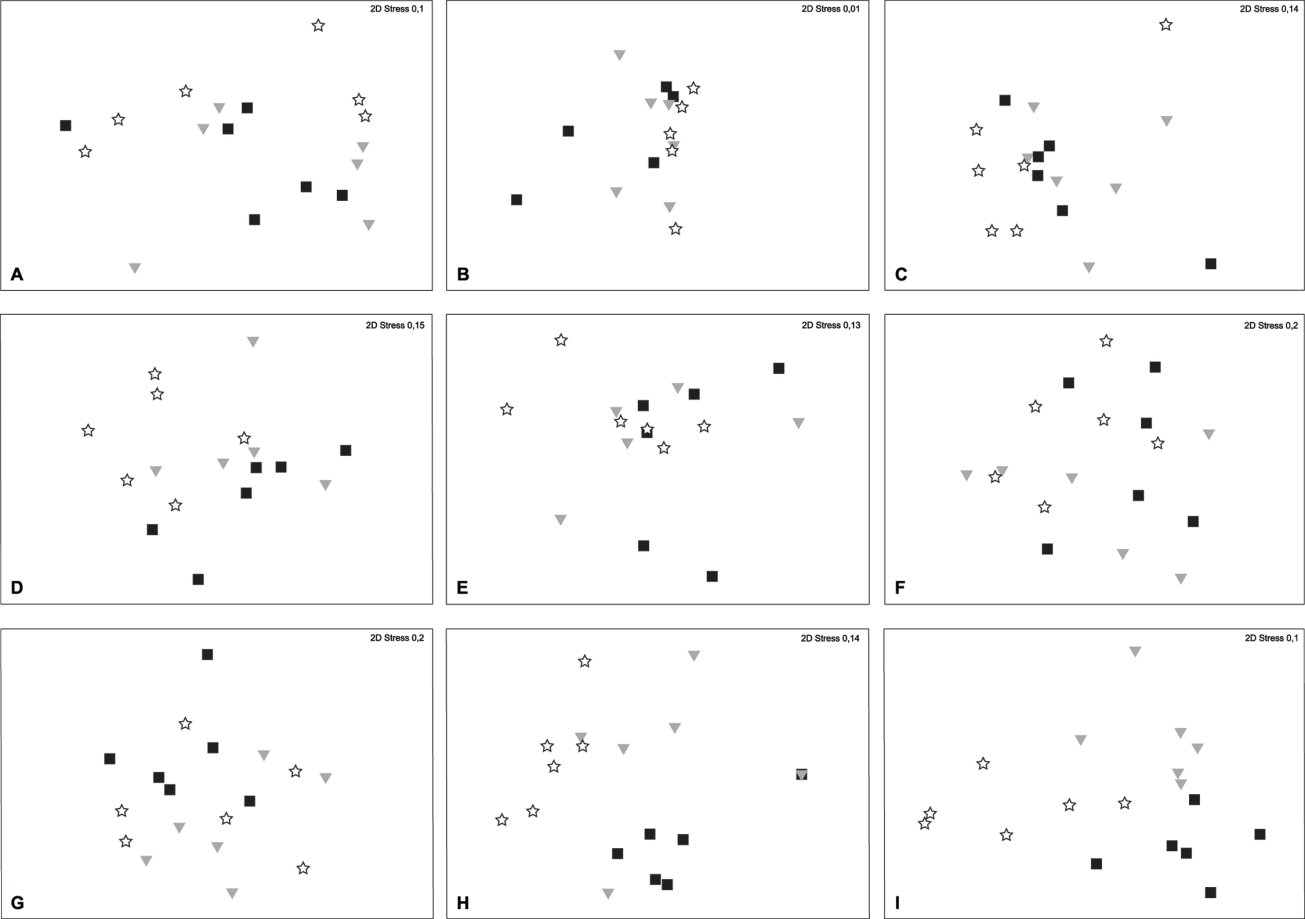
nmMDS of communities at the different size-levels. White stars label samples of the area A, gray triangles samples of the area B and black squares samples of the area C. (A) bacterial communities characterized by T-RFLP with the enzyme AluI, (B) bacterial communities characterized by T-RFLP with the enzyme BsuRI, (C) bacterial communities characterized by LH-PCR, (D) microbial eukaryotes communities characterized by T-RFLP with the enzyme AluI, (E) microbial eukaryotes communities characterized by T-RFLP with the enzyme BsuRI, (F) microbial eukaryotes communities characterized by LH-PCR in the region V1, (G) microbial eukaryotes communities characterized by LH-PCR in the region V4, (H) meiofauna communities, (I) macrofauna communities at the higher taxonomic level.

The nm-MDS representation for the bacterial community obtained by each technique is shown in Fig 3A–3C. The ANOSIM test applied on bacterial community data from the three areas gave no significant results (Table 1).

**Table 1:** Results of ANOSIM and pairwise test of data obtained from communities at the different size levels in the three areas (A, B, C). For the pairwise test, only significant results are reported.

		ANOSIM			Pairwise test	
		R	P		R	P
Bacteria	T-RFLP (AluI)	0,009	n.s.			
	T-RFLP (BsuRI)	-0,005	n.s.			
	LH-PCR (V1)	0,022	n.s.			
Microbial eukaryotes	T-RFLP (AluI)	0,201	P < 0,05	A—C	0,354	P < 0,05
	T-RFLP (BsuRI)	-0,047	n.s.			
	LH-PCR (V1)	-0,048	n.s.			
	LH-PCR (V4)	-0,017	n.s.			
Meiofauna						
	Higher taxa	0,355	P < 0,01	A—C	0,606	P < 0,01
Macrofauna	Species	0,537	P < 0,01	A—B	0,436	P < 0,05
				A—C	0,872	P < 0,01
				B—C	0,288	P < 0,01
	Higher taxa	0,570	P < 0,01	A—B	0,644	P < 0,01
				A—C	0,685	P < 0,01
				B—C	0,419	P < 0,01

#### Microbial eukaryotic community

For the microbial eukarya community, the number of detected OTUs was 16–95 with the enzyme AluI, and 13–94 with the enzyme BsuRI. For the LH-PCR technique, it was between 7 and 31 for the region V1, and 5 and 22 for the region V4. The average number of OTUs in area A, B and C obtained with the two enzymes is reported in Fig 2C. By LH-PCR technique on the two gene-sequence regions V1 and V4 the average number of OTUs is reported in Fig 2D. The difference in the percentage number of OTUs between T-RFLP and LH-PCR is 69,93% (±20.63%).

The nm-MDS representation for the community of microbial eukarya obtained by each technique is shown in Fig 3D–3G). The ANOSIM test gave a significant result (p<0.05) only for microbial eukaryotic community analyzed by T-RFLP with the enzyme AluI, the main differences being between data from areas A and C (p<0.05) (Table 1).

#### Meiofauna community

The meiofauna consisted mainly of nematodes with small percentages of other taxa (polychaetes, bivalve molluscs, amphipods, copepods, ostracods) and a total of 430 individuals were collected. The nematodes were the most abundant taxon ranging from 156 (A) to 88 individuals (B), while ostracods were present mainly in C (32) together with a few copepods (5). Polychaetes and bivalve molluscs were present mainly in A.

Regarding the meiofauna community, the medium number of taxa for each area is represented in Fig 2E. The nm-MDS representation for the meiofauna community is shown in Fig 3H. The ANOSIM test among data from the three areas gave a significant result (p<0.05), the main differences being between data from areas A and C (p<0.05) (Table 1).

#### Macrofauna community

In total, 18 macrozoobenthic species and 204 individuals were collected in the study areas. The total number of species per area ranged from 10 (B) to 12 (A, C), while total abundance ranged from 40 (B) to 97 (A). The most abundant species were the molluscs *Donax trunculus* and *Lentidium mediterraneus* and the crustacean *Diogenes pugilator*.

Regarding the macrofauna community, for each area the medium number of species is presented in Fig 2F. For the macrofauna community, on the basis of the obtained data also an assessment of the community structure at a higher taxonomic level comparable to meiofaunal ones was done. For each area the medium number of taxa was, in order for A, B and C, 2 (±1.26), 2.16 (±0.75) and 2.66 (±0.52). The nm-MDS representation for the macrofauna community at this higher taxonomic level is shown in Fig 3I. The values of ANOSIM for macrofauna are reported in Table 1.

### Pairwise comparative analysis

**Fig 4:**
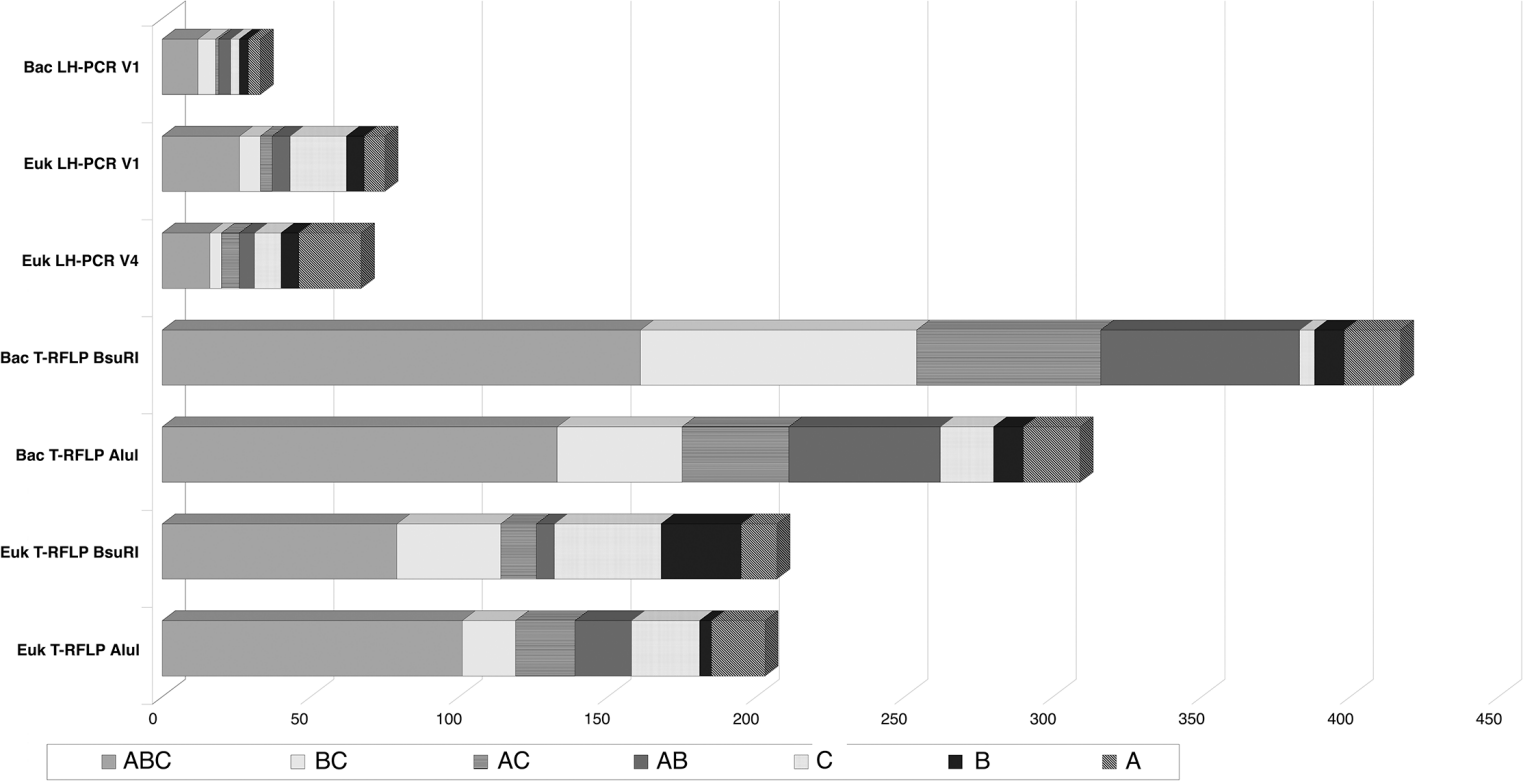
OTU's discrimination by different methods. Discrimination of the prokaryotic and eukaryotic microbial OTUs present in all the three studied areas, in two out of three areas and in each single area, by the different used molecular approaches.

A pairwise comparative analysis between the different size level communities was performed by the use of the test RELATE. For microbial communities data obtained from both techniques (T-RFLP and LH-PCR) were used and for macrofauna communities data from both taxonomic levels classification (species and higher taxa) were used. Significant results between different size-levels communities were obtained only for the comparison between meiofauna and microbial eukaryotes by LH-PCR for the V1 region (p < 0.05) and between macrofauna at the level of species and microbial eukaryotes by T-RFLP with the AluI enzyme (p < 0.05), but with low values of Rho (0.179 and 0.176, respectively). By the same test, different methods for the same community have been evaluated (concerning microbial communities). The comparison between data obtained from the use of two different restriction enzymes in T-RFLP was significant only for the microbial eukaryotic community (p < 0.01). Significant results were not obtained neither from the same comparison for bacterial communities, nor from the comparison between data of the two different region (V1 and V4) of LH-PCR on microbial eukaryotes. These two last results were probably due to the more pronounced differences in the capacity of the different techniques of discriminating among the different OTUs (Fig 4).

### Environmental parameters

The collected environmental data were square root transformed, standardized, normalized, and subjected to a Principal Component Analysis (PCA), which results are reported in S1 Fig. The stations were separated along the first axis of the model on the basis of their longitude, coarse sand percentage (ranged from 76.06% to 93.84%) and depth (ranged from 3.1 to 3.8 meters) while along the second axis on the basis of organic matter values (ranged from 0.45 to 1,74 mg g^-1^) and surface salinity (ranged from 31.7 PSU to 37 PSU) as indicator of the channel influence. These two axes explained 69.4% of the total variance. Analysis of Similarity (ANOSIM) performed on the same data gave a global R value of 0.632 (p<0.01) with main differences between area A and C (pairwise test R = 0,743, p < 0.05).

The BEST/BIO-ENV test on the different level communities gave no significant results for bacterial community as well as for microbial eukaryotes and meiofauna communities. Therefore, it was not possible to link their distribution to any of the measured environmental parameters. A correlation was only found between the distribution of macrofauna at the level of species and surface salinity together with longitude values (Rho 0.509 and p = 0.01), as well as between the distribution of macrofauna at the higher taxonomic level and organic matter values (Rho 0.610 and p = 0.01), which were well below the critical values associated with high risk of benthic disturbance from organic enrichment [27].

## Discussion

By the help of a combined approach of molecular and morphological methods, the community structures at different size-levels have been characterized and comparatively analysed. Our data confirm the necessity of using at least two different restriction enzymes in T-RFLP, in order to detect an amount of OTUs big enough to depict in a good way the diversity of the microbial communities in the natural environment (Fig 4). Moreover, in this work, for the first time, the LH-PCR method was used for the characterization of eukaryotic microbial communities. Our data show that this method can be successfully applied also to eukaryotic communities and not only to bacterial communities. Indeed, if we compare the number of obtained OTUs for T-RFLP and LH-PCR for eukaryotic and bacterial microbes, we find a lower loss of information for eukaryotes than for prokaryotes. This indicates that LH-PCR can give even a higher efficiency on eukaryotic communities with respect to its efficiency on bacterial communities. Moreover, we tested the method on two different 18S rRNA gene regions: V1 and V4. Although aware that the situation can be different in different systems, we found that analysis of V1 region could better discriminate the communities in our samples, giving a higher number of OTUs (Fig 4).

It is worthy to make some considerations more on the molecular fingerprinting approaches that we used for the characterization of microbial communities. First, as any indirect method, we are aware of some intrinsic, unmovable limits. Unfortunately, even the most recent developed method for microbial communities characterization resides on indirect techniques (*In Situ* Hybridization represents the only exception, but it is unfortunately not applicable to *a priori* unknown communities). Second, as we used both T-RFLP and LH-PCR methods for assessing the prokaryotic and eukaryotic microbial communities, we were able to determine community structures at two different levels of sensitivity. If this does not correspond exactly to a characterization performed at two different taxonomic levels, it corresponds anyway to a characterization performed at two different gene-clustering levels [28]. Of course, it is not possible to assess an exact correspondence between a certain molecular method and a precise taxonomic level of the Linnean hierarchy, but it is certain that Operational Taxonomic Units (OTUs) characterized by T-RFLP correspond to more restricted groups with respect to those characterized by LH-PCR [29]. Similarly, by the use of morphological approaches, a double characterization was also achieved for macrofauna community: thank to the relevant size of the individuals, it was indeed possible to establish the species affiliation of each individual and, consequently, also their distribution in higher taxonomic rank. Only a morphological characterization at a higher taxonomic level (phylum and subclasses) was possible for meiofauna community, due to their small size in addition to the difficulties in managing these fragile organisms without loosing some relevant parts for their identification at the species level. So, with the exception of meiofauna, we could characterize all the size levels at two different taxonomic levels. In our case study no significant changes were observed considering different taxonomic levels in the pairwise comparison of size-level communities. Nevertheless it was hypothesized that, at least in some cases, habitat filtering can have different impact at different taxonomic levels [1, 30], therefore our approach could be useful for future investigations on this aspect.

Our data seem to confirm the hypothesis that the strength of environmental determinism increases with organism size, from negligible for bacteria to stronger for microbial eukaryotes (T-RFLP with AluI), meiofauna and macrofauna respectively. Comparative analyses (R values in pairwise ANOSIM tests) of sample data from the three different areas for each size level indicate a moderate degree of sensitivity to the different environmental conditions, observed mainly between the two most distant areas (A and C). These two zones resulted more different also according to the measured environmental parameters, although in our study the only significant correlation of BEST/BIOENV test was found between macrofauna distribution and surface salinity together with longitude coordinates. This means that macrofauna communities were probably somehow influenced by their distance with respect to the mouth of the Fiume Morto, while no significant correlation was found for the other body-size community levels (i.e. the smallest ones).

Moreover, the absence of significant results in the pairwise analysis between communities of the different size-levels suggests that each community responds in a different way to the shared environmental conditions. Indeed, samples of the different size-level communities were collected at the same time, thus guaranteeing that the analyzed communities were experiencing exactly the same environmental situation. A lack of congruence in the response to environmental factors was also demonstrated by Doi and colleagues [9] even among different groups of planktonic organisms.

Farjalla and colleagues [1] showed that environmental determinism increases with organism size. This hypothesis, already proposed by other studies [31, 32], was also later confirmed [2]. The explanation probably resides in the random dispersal and shorter life cycles, typical of smaller organisms, that result in faster population dynamics [2]. Our data are in accordance with this hypothesis. Moreover, analysis of abiotic parameters demonstrate that, in this case, the environmental gradient along the studied coastline was quite weak (see S1 Fig), thus explaining why only the highest body-size level (macrofauna) was somehow sensitive to it. It could be worthy to repeat in the future the same comparative analysis with a stronger gradient, in order to verify if and to which extension such a condition can actually affect also lower size levels.

Results of the pairwise comparative analysis between the different size-level communities show a lack of congruence and the absence of any kind of relation. As already mentioned, this can be due to different responses to environmental gradients. Nevertheless, this result also assesses that community composition at one size-level could have no (or very low) influence on the community composition at other size-levels, at least in this zone, at the study spatial scales and at this time of sampling. Even if we are still far from proving a general rule, our data are, actually, in agreement with data from previous research [11]. Considering that, in the marine environment, consumers very often feed selectively according to body size of their prey, in this environment body size is surely deeply linked to the trophic level of species in the trophic chain. Therefore, an absence of relationship between the structures of different size-level communities could indicate a scarce influence of trophic interactions on the shaping of community structure. Nevertheless, Doi and colleagues [9] also suggested that the low specialization of consumers in the marine environment could explain the low effect of feeding in affecting community structure. It is worthy to remind that a consistent limit of the approach we used for microbial communities is the impossibility of discriminating between eterothrophic and autotrophic organisms. This is actually a limit of almost all the studies performed on microbial communities as a whole in the environment. Indeed, in most of the published studies aimed to clarify relationships between prokaryotes and other size-level communities, microbes are only characterized as total biomass, without any distinction between their trophisms [33, 34, 35, 36]. It is also true that, in the trophic chain, autotrophs and eterotrophs are indistinguishable edible prey for bigger organisms, independently from their metabolism.

Besides ecological considerations, obtained data give us some additional hints. Previous studies already showed that it can be very difficult to predict the total biodiversity of a system based upon a single taxonomic group [9]. Our results tell us that even characterizing the entire community composition at a certain size range cannot give any hint on community composition at other size-level. For this reason, our results suggest that even the use of the community at a certain size level as indicator for monitoring a whole ecosystem could be not always a reliable predictive tool. Therefore, the characterization of all the different size levels is clearly becoming a necessity in order to study the dynamics really acting in a system and the development and validation of reliable methods for this purpose are strongly needed.

## Supporting Information

S1 Fig PCA on abiotic data. PCA on abiotic data and samples from the three areas. The percentage of variation is 43.2 for axis 1 and 26.2 for axis 2. OS: Organic Substance; CS: Coarse Sand.
(EPS)

S1 File Supplementary information on analysis of prokaryotic and eukaryotic microbial communities. (DOC)

S1 Table Geographical coordinates of the sampling sites. (DOC)
