# Supplementary material for: A Multi Size-Level Assessment of Benthic Marine Communities in a Coastal Environment: Are They Different Sides of the Same Coin?
Source: PLoS One. 2015 Jun 15;10(6):e0129942. doi: 10.1371/journal.pone.0129942 (PMC4468208; doi:10.1371/journal.pone.0129942)
Supplement: S1 File — (DOC) [file pone.0129942.s002.doc]

***Analysis of bacterial and microbial eukaryotic communities – supplementary information.***

T-RFLP analysis.

After DNA extraction, the 16S rRNA genes were directily amplified from the DNA extracted from the first bucket, using two universal bacterial primers: 8F (5'-AGA GTT TGA T(CT)(AC) TGG CTC AG-3', Lane 1991) labelled with FAM or NED fluorochrome at the 5′ end, and the reverse R1492 (5′-GG(AGCT)(AT)AC CTT GTT ACG ACT T-3′, Lane 1991). Similarly, the 18S rRNA genes were directly amplified from the DNA extracted from the second bucket, using primers 18F6 Euk, FAM labeled, (5'-AA(CT) CTG GTT GAT (CT)(CT)T GCC AG-3', Medlin *et al.* 1988) and 18S R1492 (5'-GAA ACC TTG TTA CGA CTT-3').

The digestions with restriction enzymes were performed independently at 37 °C for 4 h using BsuRI (Fermentas, 0.2 μ/μl final concentration) and AluI (Fermentas, Canada, 0.2 μ/μl final concentration). After digestion, DNA was precipitated using sodium acetate and cold 100% ethanol to eliminate salts. For each reaction, a mix with 1 μl of size standard (GeneScan™ 500 LIZ, Applied Biosystems, USA), a maximum of 5.5 μl of sample and 13.3 μl of deionised formamide (Applichem, Germany) were prepared.

Capillary electrophoresis was performed using an Abi Prism 310 Genetic Analyzer (Applied Biosystems, USA). T-RFLP profiles were analysed using GeneScan™ analysis software (Applied Biosystems, USA).

LH-PCR analysis.

For amplification of bacterial V1 region of 16S rRNA gene, primers 8F labeled with the fluorochrome FAM (Lane 1991) and R343ND (5'-CTGCTGCCTCCCGTAG-3', modified from Vannini et al. 2004) were used as forward and reverse primers, respectively.

For amplification of eukaryotic V1 region, we used the forward primer 18F6 Euk labeled with the fluorochrome FAM. 18S_R300 (5'-TCTCCGGAATCGAACCCT-3') was used as reverse primer. For the V4 region amplification we used the forward primer TAReuk454FWD1 (5'-CCAGCASCYGCGGTAATTCC-3', Stoeck et al. 2010), labelled with the fluorochrome FAM, and the R786 Hypo (5'-CTAGGACGGTATCTGATC-3') as reverse primer.

For the region V1 PCR was performed with the annealing taking place at 60°C, while for the region V4 a touch-down PCR was performed with the annealing taking place at 60°C (5 cycles), 55°C (10 cycles) and 50°C (25 cycles).

All the products of amplification were cleaned using the EuroGold Cycle-Pure Kit (EuroClone®, Italy). Capillary electrophoresis for V1 region was prepared and performed as already described for T-RFLP analysis. Profiles were analysed using GeneScan™ analysis software (Applied Biosystems, USA).

The *in silico* analysis, carried out for region V4 using the ARB software (Ludwig *et al.* 2004), showed a lot of sequences longer than 500 bp. For this reason, the capillary elecrophoresys was carried out by the BMR Genomic laboratory of Padova, using the size standard ROX 1000 BV. Profiles were analysed using PeakScanner™ analysis software (Applied Biosystems, USA).

References.

Lane D.J. (1991) *Nucleic Acid Techniques in Bacterial Systematics*. Wiley, New York, USA, pp. 115–147.

Ludwig W., Strunk O., Westram R., Richter L., Meier H., Yadhukumar, Buchner A., Lai T., Steppi S., Jobb G., Forster W., Brettske I., Gerber S., Ginhart A.W., Gross O., Grumann S., Hermann S., Jost R., Konig A., Liss T., Lußmann R., May M., Nonhoff B., Reichel B., Strehlow R., Stamatakis A., Stuckmann N., Vilbig A., Lenke M., Ludwig T., Bode A., Schleifer K.H. (2004) ARB: a software environment for sequence data. *Nucleic Acids Research*, **32**, 1363–1371.

Medlin L., Elwood H.J., Stickel S., Sogin M.L. (1988) The characterization of enzimatically amplified 16S-like rRNA-coding regions. *Gene*, **71**, 491-499.

Stoeck T., Bass D., Nebel M., Christen R., Jones M.D.M., Breiner H.W., Richards T.A. (2010) Multiple marker parallel tag environmental DNA sequencing reveals a highly complex eukaryotic community in marine anoxic water. *Molecular Ecology*, **19**, 21-31.

Vannini C., Rosati G., Verni F., Petroni G. (2004) Identification of the bacterial endosymbionts of the marine ciliate *Euplotes magnicirratus* (Ciliophora, Hypotrichia) and proposal of '*Candidatus* Devosia euplotis'. *International Journal of Systematic and Evolutionary Microbiology,* **54**, 1151-1156.
