# Supplementary material for: A Multi Size-Level Assessment of Benthic Marine Communities in a Coastal Environment: Are They Different Sides of the Same Coin?
Source: PLoS One. 2015 Jun 15;10(6):e0129942. doi: 10.1371/journal.pone.0129942 (PMC4468208; doi:10.1371/journal.pone.0129942)
Supplement: S1 Table — (DOC) [file pone.0129942.s003.doc]

| Sampling site. | Latitude. | Longitude. |
| --- | --- | --- |
| A1 | 43° 44’ 05.0” N | 10° 16’ 27,6” E |
| A2 | 43° 44’ 05.1” N | 10° 16’ 29,6” E |
| A3 | 43° 44’ 04.0” N | 10° 16’ 26,7” E |
|  |  |  |
| B1 | 43° 43’ 46,7” N | 10° 16’ 28,6” E |
| B2 | 43° 43’ 45,0” N | 10° 16’ 28,2” E |
| B3 | 43° 43’ 37,5” N | 10° 16’ 27,5” E |
|  |  |  |
| C1 | 43° 43’ 31,5” N | 10° 16’ 30,9” E |
| C2 | 43° 43’ 28,0” N | 10° 16’ 32,4” E |
| C3 | 43° 43’ 26,3” N | 10° 16’ 32,1” E |
|  |  |  |
